# Proceedings of the Regular Meeting of the Organon Society, Boston

**Published:** 1889-03

**Authors:** S. A. Kimball

**Affiliations:** Secretary


### PROCEEDINGS OF THE REGULAR MEETING OF THE ORGANON SOCIETY, BOSTON, MASS.

The regular meeting of the Boston Organon Society was held January 10th.

Dr. Wesselhœft read from the *Organon*, beginning at paragraph 82.

Dr. Wesselhœft—I will only remark after paragraph 83, that if, after the examination of a chronic case, we give a remedy and it does not do what is expected of it, and we give a second remedy and are still disappointed, what are we to do then? In such cases I shall never forget what Dunham once told me of Bœnninghausen, with whom he had been for more than a year. He said it was the rarest thing for him to make a mistake in his first remedy, and this was especially true in intermittent fever, which was very prevalent in Munster. If he did make a mistake and the first remedy did not help, he would cross out the last examination and make a new one, starting again as if he had never seen the patient. I am reminded of this by an instance that occurred in my own practice not long ago. A patient, who had been off and on under my care since she was five years old, had a very peculiar anæmia come on after living in Paris two years. She is now twenty-two years old. After the anæmia, hysteria developed which was characterized by a left-sided paralysis, this would get better and then return. I thought the first examination was a most excellent one, but as she was going to New York for a time, I had her see a physician there, to whom I wrote a short history of the case and asked him to make a careful examination. He made a most masterly record, in which appeared an entirely new history of the beginning of the case: he found out the cause, which was a deep mental one, and I think she will now be cured. If I had crossed out my first examination a long time ago, I might possibly have come upon the same history.

We are now coming to a very important part of the *Organon*, and we must pay strict attention to every word Hahnemann has written.

Dr. Kennedy—How many physicians, homœopathic physicians, follow these directions?

Dr. Wesselhœft—Perhaps one in fifty. This part of the *Organon* is the foundation of all homœopathic prescribing; without following these directions it is impossible to prescribe accurately or homœopathically. Of course, we all slur over our work occasionally, and are not as careful as we should be. My preceptor would never let me approach him with a verbal statement, it must all be written down, if it were only a case of toothache.

Dr. Tompkins—How many patients can one see daily, if he is to make such a careful record in each case?

Dr. Wesselhœft—This is an important point. Probably none of us see more than two or three new patients daily. Now, instead of making a hasty examination, I think we would all feel better if, after studying for some time and not being satisfied, we should give them a few powders and ask them to come again in a few days, and when we could devote another hour to them.

This would be much better than to be in haste and give a wrong remedy. I know of a successful physician who never prescribes for a rose cold until he has watched the case for some time. This is much more honorable than hap-hazard prescribing. After the first prescription it is easy enough to wait.

Dr. Bell—Hahnemann used to see a great many patients daily when he was in Paris, so he is giving directions that he probably followed.

Dr. Wesselhœft—I do not think one can see many chronic patients daily. I do not think that Dr. Wilson, of London, will see more than twenty, and he devotes nearly the whole day to them.

Dr. Bell—It is a question if we should wait for each patient to go through their whole history, especially when it is of no importance. Of course, a patient should not be interrupted while relating his symptoms intelligently, but they do not always do that, and every one finds his own way of shortening an examintion while getting the essential facts.

Dr. Wesselhœft—It depends largely upon the patient, an intelligent patient can tell you in his own words. There is nothing so bad as to tell a patient to keep still and you will ask him questions. Some patients cannot express their symptoms, and you can get nothing out of them. Patients often ask how they shall report by letter, I usually tell them never to give me a symptom without its conditions.

Dr. Bell—I have here a letter which shows how a report should be made, and will read it:

"DEAR DR. BELL:—I have been suffering for the past week with a severe faceache. It does not seem to come from any special tooth, as they are none of them sore to the touch. It is on the *left* side, both upper and lower jaw, the pain in the lower jaw running forward into the chin, the jaw feels stiff when I move it, pain sharp, and going from back of upper to front of lower jaw and then back again. Outside of cheek feels *cold* to the hand, but neither hot nor cold applications relieve the pain. The pain is intermittent. It came from sailing on a very cold day, and driving in cold wind makes it much worse. It is better when I move and walk than when sitting, worse when I lie down. The pain is not the unbearable kind, but very wearing, and I find after eight days of it the world looks very cloudy."

*Diagnosis of the remedy.*

Left side: *Cham.*, *Nat-m.*, Phos., Rhus.

Whole row: Cham., Merc., Rhus, Staph.

Intermittent: Bell., Bry., Cham., Coff., Chin., Merc., Nux-v., Puls., Rhus, Sil., Staph., Sulph.

Caused by cold wind: Acon., Puls., Rhus, Silicea.

Worse from cold wind: *Bell.*, Hyosc., *Nat-m.*, Nux-v., Sabin., Sil., Staph., Sulph.

Better moving: Puls., Rhus.

Worse sitting or lying: *Ars.*, Bell., Bry., *Cham.*, Hyos., Ign., Merc., Nux-v., Phos., *Puls.*, *Rhus*, Staph., Sulph.

As it was caused by the cold wind, and because she was of a mild, gentle disposition, I sent Puls., and in five days received the following:

"Dear Dr. Bell:—My face has been much better, although I have felt it a little at night.

"Yesterday I ventured out, and the cold air brought back the pain, not at the time, but it came in the night and continues this morning. The pain is worse or centres in the jaw-joint near the ear, running down through the jaw and up into the temple. The pain is sharp but does not last long at a time. The upper part of jaw, and the joint feel *numb* all the time.

"It is still entirely on the left side, other symptoms the same as when I wrote last, indeed, the only change seems to be that it has settled higher up in the jaw, the feeling of numbness in the joint is very marked, although it is *not stiff* when I move it."

On account of the numbness I sent a dose of Mezereum, which speedily removed the whole trouble.

Dr. Winn—Perhaps it is not exactly appropriate, but this report of the toothache reminds me; I would like to ask about amalgam fillings in teeth.

Dr. Wesselhœft—We are told there is no appreciable oxidation of the mercury in an amalgam filling, but I thing there is enough to affect the patient, and in affections of the mucous membranes I have patients take out their amalgam fillings and take off their woolen under-clothing. I was first led to see the harmful effects of amalgam fillings by an ulcer on the tongue of a patient, a number of years ago. It was a terrible, punched-out ulcer with high edges, and had been going on for some time. It was very obstinate; I treated it for several weeks without much change. At last I discovered that this ulcer fitted right over an amalgam filling of a built-up tooth. I had this amalgam taken out and a soft filling substituted, then the ulcer healed in a surprisingly short time. The amalgam filling had been in many years.

Dr. Tompkins—How about the red rubber plates for false teeth; is there not cinnabar in such plates?

Dr. Wesselhœft—I always have them taken out if possible, and black rubber substituted.

Dr. Davis—How is it best to keep records?

Dr. Wesselhœft—My father used to have a large folio, and, as he wrote a very small hand, he could use one page to a case. I used to use separate slips; they are very convenient when you have to visit chronic cases. Now I use medium-sized books, indexed, one volume from A to J, the other K to Z. The important point is to keep a record that you can refer to, for it is very reassuring to a patient to have you refer back to a record made five, ten, or twenty years before, and compare his present condition to the previous one. Bœnninghausen's practice was altogether an office one, and he kept his records in large folios.

Dr. Hastings—I find after a patient has been carefully examined that there are often symptoms given that we cannot find in the *Materia Medica.*

Dr. Wesselhœft—We often cannot find the symptoms, but we can find the conditions.

Dr. Davis—Are not the concomitants more important than the symptoms?

Dr. Kennedy—We often find patients giving symptoms in regard to their eyes and ears, for instance, that we cannot find in the books.

Dr. Wesselhœft—That is where the modalities come in, and we have to go by them.

Dr. Tompkins—Provers were not always questioned as carefully as patients are, except by Hahnemann.

Dr. Wesselhœft—A good way to do is to ask in the line of a remedy if you get a cue. That is, ask some of the leading characteristics of a remedy that the patient's answer may suggest, and if you find that his case does not correspond to that remedy, you can try another.

Dr. Dike—Are we not apt to be biased in favor of remedies that we know best?

Dr. Wesselhœft—Yes, I think we are, and we must be careful not to ask leading questions.

Dr. Kennedy—A point that is sometimes overlooked is, that the peculiarities of a patient in health are often not taken into account enough. I think we are often led to the remedy by the ameliorations or aggravations of the patient's general condition when he calls himself well.

Dr. Wesselhœft—We often know when a patient comes in and before he has talked much that some remedies are excluded.

Dr. Bell—The making up of a record stamps the physician as a careful man; the patient feels better for it, and it gives him great confidence, particularly if he comes back after five or ten years and finds that you still have it.

Dr. Wesselhœft—Patients can be held by a fortnightly or monthly examination of which a record is carefully taken, but you cannot remember without a record.

Dr. Hastings—Patients often say they are no better, but on going over the last examination you find they are very much better.

Dr. Wesselhœft—We often hear chronic complainers say: "I don't know that I am any worse." That always means that they are better. We can tell by the record if we get an aggravation, and those aggravations that last a long time are very important.

Dr. Tompkins—Patients should be educated in regard to hay fever, etc., so that they will not try to obtain temporary relief from patent medicines and such things.

Dr. Wesselhœft—How much relief do they get? Of course, they must be educated. I think if you explain to patients the necessity of their coming to you before the attack comes on, they will understand it.

Dr. Bell—I believe if Beecher had been treated homœopathically for his hay fever instead of running all over the country, he would not have died of apoplexy.

Dr. Wesselhœft—Apoplexy is always of a deeply psoric origin.

Dr. Tompkins—Could not the people be educated by popular tracts of the right sort?

Dr. Wesselhœft—I think it would be a good idea to have a popular treatise on Hahnemann's psoric theory and the metastasis of disease. It would have to be carefully done, but would be an important factor in educating the people up to these things.

Dr. Winn—Do not the allopaths admit of chronic diseases?

Dr. Wesselhœft—They talk of a diathesis, the word is getting more and more common. Syphilitic diathesis, they are up on that now; that is because it is more acute than the other miasms, sycosis being less acute and psora least acute. They will come to these, however; look at syphilis, what does it not do now? What did it do forty years ago? The next thing will be no local treatment for the original cause.

Dr. Bell—It is a good idea to tell patients that such things are constitutional.

Dr. Wesselhœft—Don't tell them that the suppression of such things by local measures will produce the same thing internally, but it will disarrange the internal organs.

Dr. Tompkins—I think we can tell patients that they will be better after an acute disease when it has been carefully prescribed for and so cured.

Dr. Bell—I have now under my care a singer with a bad cold; she will probably get over it in a week, but she will be better after the cold is cured, and I told her that she would not take cold so much after one or two of them had been cured homœopathically.

Dr. Wesselhœft—Most professional singers are homœopathists; they know what helps them. I tell them they must not take cold, that shows that something is the matter with them. It is better for them to take the remedy for a cold, even if they recover no quicker than without it; it will prevent their catching cold so easily again. Colds often indicate the psoric remedy, but when a patient is under treatment for a chronic disease, and under the influence of a remedy, I never prescribe for a cold, always give them sugar, the cold often shows what the remedy is doing.

Dr. Tompkins—The allopaths never think of the future health of a patient.

Dr. Wesselhœft—That shows the beauty of Hahnemannian philosophy. A patient under allopathic treatment for mucus in the throat, will be sent to an eye doctor for anything the matter with her eye, or to an ear doctor for her ear, and all these may be prescribing for her together. Hahnemann's idea is so much more beautiful than such cobbler's work, and after we have met such things and have overcome them, then we can realize something of the beauty and grandeur of Homœopathy.

Adjourned to January 24th.

---

### Regular Meeting, January 24th.

Dr. Wesselhœft read from the *Organon*, beginning at paragraph 92.

Dr. Bell—We may find a patient under the influence of Morphine with contracted pupils, it may be necessary to interfere. When such drugs are stopped, we are going to get their secondary effects, and these we have to meet without waiting for them to pass off.

Dr. Cobb—Would the effect on the system be better to allow the drug effect to pass off without medicine? Now I had a case before coming here this evening, where the symptoms were due to over-indulgence in coffee. Ignatia seemed indicated, and I gave it.

Dr. Wesselhœft—I think in such cases if we find a remedy indicated we should give it. But, if you have given a carefully selected remedy in a chronic disease, and the patient comes in with symptoms from some indiscretion in diet, it is much better to let it pass off than to interfere with another remedy; such is my experience.

Dr. Bell—You may be called to a case of typhoid fever where the temperature has been kept down by antipyrin, and you may have a drug effect when the antipyrin is stopped. Dr. Duke, in his book, speaks of a man with a congestive headache whose feet were put in hot water, and he was much relieved. But, later, the congestion returned with much greater severity, resulting in unconsciousness and death. That was not a drug effect, but it was a secondary effect of applying the hot water to the feet.

Dr. Wesselhœft—Note seventy-nine is very important, particularly that part concerning mental causes and influences. It is a most important thing in chronic diseases.

Dr. Bell—How do you get the confidence of the patients so that they will tell you their mental symptoms?

Dr. Wesselhœft—I usually tell them, if I have a suspicion that there is a mental cause in the case, that this examination is entirely different from any other examination they ever had, that often mental influences have a serious effect upon a patient, and that the examination is entirely confidential, and then I ask them if they have had great griefs or troubles. I often feel in the examination of a patient that there is something at the bottom of the case, some mental disturbance. I had an instance to-day in a case that I have been treating three months without success. I have had a suspicion that some mental trouble was at the bottom of it all, so I obtained her confidence and found an enormous mental history. We often know by the way a patient reports that there is a mental cause for the trouble.

Dr. Bell—Sometimes they will conceal the history of mental grief, sometimes they will tell it.

Dr. Wesselhœft—I have found that infectious diseases are more often concealed than emotional ones. I recollect a case I treated a long time without success when a friend of the patient said one day, "You will never get her well until she gets over her jealousy." The next time the patient came in I found on careful inquiry that she was very jealous of her husband, with no reason for it. This note is very instructive, especially in its list of emotions.

Dr. Bell—All such causes are practically overlooked by the old school.

Dr. Wesselhœft—How much Ignatia, Hyoscyamus, Staphisagria, Phosphoric acid, and such remedies help us, and by carefully comparing them we are often lead to the remedy necessary for the cure.

Dr. Tompkins—What are the mental indications for Phosphoric acid?

Dr. Wesselhœft—A condition of apathy, resulting from mental griefs and such causes. How many griefs have been helped by Ignatia!

Dr. Dike—Can we always trace the connection between the cause and the result? I have a patient, the insanity of whom I think is due to a fall, but Arnica does not help.

Dr. Wesselhœft—The fall may not have been the cause, but many complaints date from the time of a fall, so with mental emotions they may be a cause. Of course, the majority of cases are not traced to mental causes, but when it is so, it is of great importance.

Dr. Bell—I had a case of a young man suffering from delusional insanity, he could not sleep, and he had been to a so-called homœopath who had given him remedies, also sleeping powders without avail. I found on careful inquiry that he had had a severe disappointment in love. He was very suspicious, thought he was being followed, fearful of bodily injury, sleepless. He got Hyosc., and in a short time was practically restored.

Dr. Tompkins—Do you give the preference to the drug covering the symptoms present, or to the drug covering the cause?

Dr. Wesselhœft—I am apt to question in the line of the drug covering the cause, such as griefs, anger, etc. If I find nothing in the line of one remedy I drop it and try another, if I find nothing after trying several remedies I then make up my mind that the element of grief or anger, etc., had nothing to do in the causation of the case, and take the present symptoms as the only factors. How many times it happens that after writing down a whole list of symptoms you find out that all this trouble has dated from a certain mental shock; then you can go back and supplement your examination by asking for symptoms in the line of certain remedies.

Dr. Bell—I think the cause sometimes overrides everything. I recall a case of colic where Bell. was indicated but did not help. I found the patient had been very indignant and the colic followed. I then gave him Coloc., which relieved him at once. It was not a Coloc. colic with doubling up and relief from pressure, etc., but the cause pointed to the remedy.

Dr. Tompkins—I recall a case of so-called gall-stone colic, where the patient had had several attacks treated by Morphine by a so-called homœopath. I found out that these attacks always came on after domestic disturbances. I gave her Staph., and she has never had an attack since, that was a year or more ago.

Dr. Wesselhœft—Hahnemann's genius in this particular discovery has always been to me most wonderful in my own experience, that he should have found not only remedies for mental emotions, but that these emotions of themselves are the exciting cause of chronic ailments shows a wonderful genius. It is easy for us to follow him. We know that an angry mother's milk will kill a child, and we can find no microbes or anything else in the milk to account for it, but to have discovered that chronic troubles date from griefs and mental emotions, shows a power of observation never before equaled. Ignatia and Nux vomica both contain the alkaloid Strychnia, and are opposites in temperament. Dr. Hering once told me that Hahnemann did not know that Ignatia contained Strychnia, and yet Hahnemann said not to follow Ignatia by Nux because they were so nearly allied. In regard to note eighty, the symptoms before dining and after any function are of the utmost importance.

Dr. Kennedy—I have a case now that illustrates that point, a case of mania in a young lady who says she has lost her mind, says she lost it a year and a half ago. She can talk rationally enough before strangers but is always sad before her family; says she takes no interest in anything. One of the things that suggested the remedy was the general disturbance before menstruation—pains, headache, etc.—all of which were relieved by the flow, another thing was that she was always worse after sleep; these led me to the remedy, from which I hope to get a good result.

Dr. Wesselhœft—That is very nice. I hope to hear how it comes out. That is the way that remedies often have to be selected; we find the indications for the remedy in an entirely different sphere from that for which they expect to be treated. Another thing occurs to me about giving a remedy for difficult menstruation. If the menses are at hand, wait until menstruation is over before giving the remedy. The most mischief is done by giving a remedy at the time of an agonizing dysmenorrhœa—that is, if the patient is under treatment. The curing must be done in the intervals between the menses; such has been my experience with most of the cases that have been relieved. Hahnemann speaks particularly of menstruation as an important consideration in the selection of a remedy.

Dr. Kennedy—In regard to Section ninety-five, I think we often find at the second or third examination that patients have troubles that they have never mentioned, because they have had them so long and have become so accustomed to them that they think it is no use to bother about them.

Dr. Wesselhœft—This is a very important paragraph; we often have just such cases to deal with. There is another class of patients, and usually a very intelligent class, who say they do not wish to complain any more than they are obliged to; they think it is wicked to complain too much. But when you explain to them and show that often things they think of the least importance are of the greatest value in selecting a remedy, they realize the importance of the symptoms and often help you very much.

Dr. Tompkins—The most unimportant thing in their opinion often leads to the remedy.

Dr. Bell—Yes, and you often get that very important thing just as they are going out of the door.

Dr. Wesselhœft—These things are usually of no importance in the diagnosis of the disease, the homœopathic physician has to go to work in an entirely different manner in the diagnosis of his remedy.

Dr. Bell—There is still another class of patients who do not want you to care anything for any other symptoms except what they come for. Those are the symptoms they wish cured, and they do not want you to ask about any others.

Adjourned to February 7th.

S. A. Kimball, *Secretary.*

